# Probiotic *Enterococcus mundtii* Isolate Protects the Model Insect *Tribolium castaneum* against *Bacillus thuringiensis*

**DOI:** 10.3389/fmicb.2017.01261

**Published:** 2017-07-07

**Authors:** Thorben Grau, Andreas Vilcinskas, Gerrit Joop

**Affiliations:** ^1^Institute for Insect Biotechnology, Justus-Liebig-University GiessenGiessen, Germany; ^2^Department of Bioresources, Fraunhofer Institute for Molecular Biology and Applied EcologyGiessen, Germany

**Keywords:** *Tribolium castaneum*, probiotics, *Enterococcus mundtii*, *in vivo* model, antimicrobial, *Bacillus thuringiensis*

## Abstract

*Enterococcus mundtii* strains isolated from the larval feces of the Mediterranean flour moth *Ephestia kuehniella* show antimicrobial activity against a broad spectrum of Gram-positive and Gram-negative bacteria. The *in vitro* probiotic characterization of one isolate revealed a high auto-aggregation score, a hydrophilic cell surface, tolerance for low pH, no hemolytic activity, and susceptibility to all tested antibiotics. We used the red flour beetle *Tribolium castaneum*, an established model organism, for the *in vivo* characterization of one probiotic *E. mundtii* isolate from *E. kuehniella* larvae. *Tribolium castaneum* larvae were fed orally with the probiotic isolate or the corresponding supernatant and then infected with either the entomopathogen *Bacillus thuringiensis* or *Pseudomonas entomophila*. Larvae exposed to the isolate or the supernatant showed increased survival following infection with *B. thuringiensis* but not *P. entomophila*. Heat treatment or treatment with proteinase K reduced the probiotic effect of the supernatant. However, the increased resistance attracts a fitness penalty manifested as a shorter lifespan and reduced fertility. *T. castaneum* has, pending on further research, the potential as an alternative model for the pre-screening of probiotics.

## Introduction

All animals are associated with a diverse microbial community that promotes their health (McFall-Ngai et al., 2013; Sommer and Bäckhed, 2013). Any disruption to the population of gut microbiota caused by antibiotics or immune system deficiency therefore reduces the fitness of the host (Lozupone et al., 2012; Modi et al., 2014) whereas the administration of probiotic bacteria can increase fitness (Buffie and Pamer, 2013). Probiotics are defined as “live microorganisms, that when administered in adequate amounts, confer a health benefit on the host” (Hill et al., 2014).

Probiotics have many applications, including the optimization of growth and survival in animal species used for aquaculture and agriculture (Chaucheyras-Durand and Durand, 2009; Pandiyan et al., 2013), and the prevention or treatment of gastrointestinal tract infections in humans (Deshpande et al., 2010; Kotzampassi and Giamarellos-Bourboulis, 2012). Probiotics have several mechanisms of action, including the production of antimicrobial compounds, the inhibition of virulence genes, the enhancing of epithelial barrier functions or the stimulation of the host immune system (Oelschlaeger, 2010). The best-studied microorganisms with probiotic activity are the bifidobacteria, lactobacilli, enterococci and yeasts (Varankovich et al., 2015). The genus *Enterococcus* (order lactobacillales) is a controversial group that contains both probiotic strains (Hosseini et al., 2009; Strompfová and Lauková, 2009; Al Atya et al., 2015) and pathogenic strains (Higashide et al., 2005; Martin et al., 2005; Gaspar et al., 2009). Enterococci produce organic acids, hydrogen peroxide and up to four different classes of enterocins (Franz et al., 2007), supporting the call for a legislative framework for probiotics (Papadimitriou et al., 2015).

The adult gut microbiota of mammals is essential for the development of the immune system in the offspring (Round and Mazmanian, 2009; Gomez de Aguero et al., 2016). Animals have evolved different ways to transfer beneficial microbes to their offspring, e.g., female mammals can transfer their own beneficial microbes through milk during lactation (Jost et al., 2014). Birds can transfer their microbes via the eggshell or regurgitation (Godoy-Vitorino et al., 2010; Ruiz-De-Castañeda et al., 2011; Kohl, 2012), and insects can transfer beneficial microbes by trophallaxis or coprophagy (Koch and Schmid-Hempel, 2011; Engel and Moran, 2013; Brune and Dietrich, 2015). Insect feces are not only relevant for microbial transmission but they also fulfill a protective function (Rosengaus et al., 2013; Diehl et al., 2015). This is particularly relevant in the case of storage pests, which defecate and live in the same environment.

*In vitro* assays for the screening of probiotic microorganisms typically test for antimicrobial activity, microbial colonization, and safety. *In vivo* assays are also recommended, because probiotics can negatively affect certain host species, as observed in honeybees (Ptaszyńska et al., 2015) and in humans (Besselink et al., 2008). The regulatory framework governing probiotics varies in different countries, making it difficult to find standardized methods (Baldi and Arora, 2015). *Caenorhabditis elegans*, *Drosophila melanogaster*, and *Galleria mellonella* are currently used for the preclinical screening of probiotics, because they are suitable for large-scale screening therefore reduce costs compared to mammalian models (Papadimitriou et al., 2015; Vilela et al., 2015). Invertebrates are also more suitable for ethical reasons and with respect to the 3Rs strategy (Russell and Burch, 1959). *Tribolium castaneum* is a well-established insect model organism, which is easy and inexpensive to rear in the laboratory. *T. castaneum* is currently used as a model for infection, for transgenerational effects and for the screening of drugs (Zou et al., 2007; Roth et al., 2010; Milutinović et al., 2014; Bingsohn et al., 2016) but not thus far for the screening of probiotics. This model insect is well-suited for screening based on functional genomics because it benefits from a sequenced genome (Tribolium Genome Sequencing Consortium et al., 2008), well-established RNA interference (RNAi) techniques (Bucher et al., 2002; Knorr et al., 2013), a recently established CRISPER/Cas system for gene knockout (Gilles et al., 2015), and the availability of transcriptome datasets generated under various conditions (Park et al., 2008; Altincicek et al., 2013; Dippel et al., 2014). It is also possible to generate axenic strains, which are useful for the analysis of probiotic microbes in isolation (Futo et al., 2016).

Here, we report the *in vitro* probiotic characterization of *Enterococcus mundtii* isolates, sourced from the feces of the Mediterranean flour moth *Ephestia kuehniella*, a common storage pest. We also investigated the *in vivo* protective role of one *E. mundtii* isolate and the corresponding supernatant by oral administration to *T. castaneum* before challenging the beetles with different entomopathogenic bacteria. We conclude that *T. castaneum* is suitable as a high-throughput screening platform for the *in vivo* testing of potential probiotics.

## Materials and Methods

### Insect Rearing

*Ephestia kuehniella* larvae (provided by the Julius Kühn-Institut, Berlin, Germany) were kept in glass jars at room temperature in darkness, and were fed on wheat grains (Alnatura, Bickenbach, Germany). *T. castaneum* Cro1 beetles collected in 2010 (Milutinović et al., 2013) were maintained on a heat-sterilized standard diet of wheat flour (type 550, Alnatura, Bickenbach, Germany) and 5% brewer’s yeast at 32°C and 70% humidity in darkness.

### Bacterial Isolation and Identification

Bacteria were isolated from the feces of *E. kuehniella* larvae. Feces were plated with a spatula tip onto casein soya agar and incubated for 48 h at 30°C. Randomly chosen colonies were re-streaked on de Man, Rogosa and Sharpe (MRS) agar to obtain pure isolates. Isolated strains were used to prepare glycerol stocks at -20°C. All isolates were identified as strains of *E. mundtii* based on 16S rDNA analysis. Bacterial DNA was amplified using primers p8FPL 5′-AGT TTG ATC CTG GCT CAG-3′ and p806R 5′-GGA CTA CCA GGG TAT CTA AT-3′ (Relman et al., 1992) yielding a product of ∼800 bp. The following PCR program was used: 94°C/7 min; 35 cycles at 94°C/60 s, 55°C/60 s, 72°C/60 s; and a final extension step at 72°C/10 min. PCR products were separated by 1% agarose gel electrophoresis and stained with SYBR Safe (Thermo Fisher Scientific, Waltham, MA, United States). Prior to sequencing (Macrogen Europe, Amsterdam, Netherlands), the PCR products were purified using a DNA purification kit (Macherey-Nagel, Düren, Germany). Sequences were deposited at GenBank (Supplementary Table S1). A bacterial phylogenetic tree was created using the neighbor-joining method (Saitou and Nei, 1987) by aligning the 16S rDNA sequences against known sequences in the NCBI database using MAFFT v7 (Katoh and Standley, 2013).

### Antimicrobial Characterization

The *E. mundtii* isolates were first screened using the agar spot on lawn technique (Schillinger and Lücke, 1989) with the following modifications for antimicrobial activity. Overnight cultures of the isolates grown in MRS medium at 30°C were spotted (7 μl) onto 0.7% MRS agar plates and incubated at 30°C for 24 h under aerobic conditions. The indicator bacteria (**Table 1**) comprised an overnight culture mixed with 1% lysogeny broth (LB) agar at a final concentration of 1 × 10^5^ cells ml^-1^. Previously spotted isolates were overlaid with 10 ml of the indicator bacteria. After complete solidification of the upper layer, the plates were incubated for an additional 16 h at 30°C under aerobic conditions. Inhibition was scored positive if the radius of the zone of inhibition around the colonies of the isolates was 1 mm or larger. We carried out three replicates per isolate.

**Table 1:** Agar spot on lawn test with *E. mundtii* isolates against indicator bacteria.

	Isolated *E. mundtii* strains														
	
Indicator strains	1	2	3	4	5	6	7	8	9	10	11	12	13	14	15
*Bacillus thuringiensis* (DSM 2046)	+	++	++	++	++	++	++	++	++	++	++	++	++	++	++
*Escherichia coli* (strain D 31)	+	+	+	+	+	++	+	+	+	+	+	+	+	+	+
*Klebsiella terrigena* (DSM 2687)	+	++	++	++	++	+	+	+	+	+	+	+	++	+	++
*Listeria grayi* (DSM 20601)	+	+	+	-	+	+	-	-	+	+	-	-	-	+	+
*Listeria innocua* (DSM 20649)	+	+	+	-	-	+	-	-	-	+	-	-	+	+	+
*Listeria seeligeri* (DSM 20751)	+	-	-	+	+		-	-	-	+	-	-	+	+	+
*Micrococcus luteus* (DSM 20030)	+	++	++	++	++	+	++	++	+	++	++	++	++	++	++
*Pseudomonas aeruginosa* (DSM 50071)	+	+	-	+	-	-	+	-	-	++	-	+	-	+	+
*Pseudomonas entomophila* (DSM 28517)	+	++	++	+	+	+	+	+	+	+	+	+	++	+	++
*Salmonella subterranean* (DSM 1620)	+	-	+	+	+	+	-	-	-	+	-	-	+	+	+
*Staphylococcus aureus* (DSM 2569)	+	+	-	-	-	-	-	-	-	-	-	-	-	+	+


*Positive inhibition was scored if the radius of the zone of inhibition around the colonies of the isolates was 3–5.99 mm (++) or 1– 2.99 mm (+) and negative inhibition (-) was scored if the zone was 0–0.99 mm. Scores are based on three replicates. The DSM number refers to strains obtained from the DSMZ strain collection, Braunschweig, Germany.*

Inhibitory substances from the isolates were further characterized by the agar well-diffusion assay (AWDA) (Tagg and McGiven, 1971) with the following modifications. *E. mundtii* isolates were grown under aerobic conditions for 24 h at 30°C in MRS medium. The cell free supernatant (CFS) was obtained by centrifugation (3200 × *g*, 4°C, 15 min), adjusted to pH 6.5 and passed through a 0.22-μm filter (Carl Roth, Karlsruhe, Germany). Afterward the CFS was concentrated 100-fold, mixed with ethyl acetate (1:1), and shaken vigorously for 1 h. The mixture was stabilized for a further 1 h and then the aqueous phase was removed. The solvent was concentrated under reduced pressure in a rotational evaporator (Büchi Labortechnik AG, Switzerland). To reduce the effect of proteinaceous compounds, 2 ml concentrated CFS was mixed with 80 μl 25 mg ml^-1^ proteinase K (Sigma-Aldrich, Taufkirchen, Germany) for 1 h at 37°C. To reduce the effect of H_2_O_2_, 2 ml concentrated CFS was treated with 40 μl 2 mg ml^-1^ catalase (Sigma) for 1 h at 37°C. Finally, the heat stability of CFS was tested by heating for 10 min at 98°C. For the AWDA, 40 μl of CFS treated in one of the three ways described above was transferred to 5-mm wells in 1% LB agar plates containing 10^5^ indicator bacteria ml^-1^. The plates were incubated at 30°C for 16 h. The zones of inhibition were measured in mm using a digital caliper, with three replicates per isolate. Sterile concentrated MRS medium was used as a negative control.

### Probiotic Characterization

Auto-aggregation correlates based on bacterial adhesion to host cells were determined as described (Del Re et al., 2000; Al Kassaa et al., 2014). *E. mundtii* isolates were grown in MRS medium for 16 h at 30°C. Cells were harvested by centrifugation (3200 *g*, 4°C, 15 min) and washed twice with sterile phosphate buffered saline (PBS; pH 7), re-suspended in PBS and adjusted to 10^8^ cells ml^-1^. The cell suspension (4 ml) was mixed by vortexing and incubated at room temperature for 24 h. After 5 and 24 h, 100 μl of the upper phase was removed and the absorbance at 600 nm was measured. The percentage of auto-aggregation was calculated as follows:

$$\%\;autoaggregation=[\frac{\left({OD}_{1}-{OD}_{0}\right)}{{OD}_{1}}]\times 100$$

where OD_1_ is the optical density at 5 or 24 h and OD_0_ is the optical density at time point zero. This experiment was performed in triplicate.

To detect bacterial adhesion to solvents, a hydrophobicity assay was carried out as previously described (Rosenberg et al., 1980; Al Kassaa et al., 2014). The bacterial suspension was prepared as described for the auto-aggregation assay, and 3 ml of the suspension was mixed with 1 ml xylene (Carl Roth) by vortexing for 2 min. The aqueous phase was removed after incubating the mixture for 10 min at room temperature, and after incubation for a further 2 h at room temperature the absorbance was measured at 600 nm. The percentage of hydrophobicity was calculated as follows:

$$\%\;hydrophobicity=[\frac{\left({OD}_{1}-{OD}_{0}\right)}{{OD}_{1}}]\times 100$$

where OD_1_ is the optical density after 2 h and OD_0_ is the optical density before adding the xylene. This experiment was performed in triplicate.

Isolate tolerance to low pH was determined as described (Hosseini et al., 2009) and reveals how well the bacteria can survive under harsh gut conditions. An overnight culture was washed twice with PBS and then resuspended in PBS acidified to pH 2, 3, and 4, respectively. After incubating for 3 h, the bacteria were plated on LB agar in order to count the number of colony-forming units (CFUs).

### Safety Characterization

Hemolytic activity was determined by spotting 7-μl overnight cultures of *E. mundtii* onto Columbia sheep blood agar plates as a required safety assay (bioMérieux, Marcy-l’Étoile, France). The plates were incubated for 48 h at 30°C. The presence of clear zones around the colonies indicated hemolytic activity (Abriouel et al., 2008). *Staphylococcus aureus* (DSM 2569) was used as a positive control. The experiment was performed in triplicates.

The sensitivity of the isolates to antibiotics was determined by performing a broth microdilution assay in 96-well plates (Wiegand et al., 2008). The following antibiotic concentration ranges (μg ml^-1^) was used: ampicillin (0.25–128), rifampicin (0.25–128), erythromycin (0.25–128), kanamycin (2–256), ciprofloxacin (0.25–128); fosfomycin (0.25–128), streptomycin (2–256), tetracycline (0.25–128), and vancomycin (0.25–128). Isolates were grown in LB medium at 30°C overnight and the bacterial suspension was adjusted to 10^6^ cells ml^-1^. A 50-μl aliquot of the suspension was then mixed with 50 μl of the antibiotic solution in a microtiter plate well and incubated for 16 h at 30°C. The minimal inhibitory concentration (MIC) was defined as the lowest concentration that inhibits visible growth.

### *In Vivo* Characterization Following Entomopathogenic Challenge

For the survival assay, 15 glass jars containing approximately 100 adult *T. castaneum* (∼1 month old) were transferred to 100 g flour for oviposition. On the third day after oviposition, the eggs were removed by sieving through a 250-μm mesh (Retsch, Haan, Germany). The eggs collected from all jars were mixed, and ∼400 eggs were transferred to the different probiotic diets (see below) each on 150-mm Petri dishes. After 8 days on these six diets, 96 larvae of similar size were transferred individually to the three challenged diets, one per well of a microtiter plate. A total of 1728 larvae were used in this experiment. The plates were sealed with transparent adhesive foil and punctured with small holes for air supply. Survival was monitored daily for 7 days. The experiment was carried out under standard rearing conditions (32°C and 70% humidity).

### Diet Preparation

For the preparation of the different probiotic diets, 0.15 g ml^-1^ of flour (type 405, Alnatura, Bickenbach, Germany) supplemented with 5% brewer’s yeast was mixed with: (1) sterile MRS medium as a negative control; (2) 5 × 10^9^ cells ml^-1^ of *E. mundtii* 1 isolate, grown in MRS medium at 30°C overnight, washed with PBS twice, centrifuged (3200 × *g*, 4°C, 15 min) and re-suspended in MRS medium; (3) crude CFS from the same isolate; (4) CFS heated at 98°C for 20 min; (5) CFS treated with 4 ml proteinase K (25 mg ml^-1^) for 1 h at 37°C; or (6) CFS adjusted to pH 7. The diet was poured into Petri dishes either (150-mm diameter = 100 ml or 90 mm diameter = 20 ml), dried at 37°C and shredded.

The immune challenged diet containing *Bacillus thuringiensis* (DSM 2046) was prepared as previously described (Milutinović et al., 2013). We added 0.15 g flour with 5% brewer’s yeast to 1 ml of the bacterial suspension, and adjusted the cell density to 5 × 10^9^ cells ml^-1^. The diet containing *Pseudomonas entomophila* (DSM 28517) was prepared by growing *P. entomophila* overnight at 30°C in LB, centrifuging the suspension (3200 × *g*, 4°C, 15 min) and washing the pellet twice in PBS before re-suspending in PBS. The cell density was adjusted as described for *B. thuringiensis*. PBS mixed with flour and yeast was used as negative control. We transferred 40 μl of the suspension to each well of a 96-well plate and dried the plates at 37°C overnight.

### *In Vivo* Characterization of Fitness Parameters

For the longevity experiment, 10 glass jars containing ∼100 adult beetles (∼1 month old) were transferred to 100 g flour for oviposition. After 24 h, the eggs collected from all jars were mixed and ∼100 eggs were transferred to the MRS, CFS or *E. mundtii* probiotic diets prepared as described above on 90-mm Petri dishes. Five replicates per diet were incubated under standard conditions. Longevity was measured using a thermotolerance assay at 42°C (Grünwald et al., 2013). Twenty-five age-controlled beetles of mixed sex were transferred after 5 days on the standard diet to the same probiotic diet used to rear the larvae. Survival was recorded daily, and dead beetles were removed from the Petri dishes.

The effect of the probiotic diets (MRS, CFS and *E. mundtii*) on the fitness of *T. castaneum* was determined by measuring the reproductive success of the beetles. Age-controlled virgin beetles were obtained as described for the longevity experiment with the additional sexing of the pupae. Five days after eclosion, virgin beetles (five of each sex, each diet with five replicates) were allowed to mate for 24 h on 10 g of flour in Falcon tubes covered with breathable tissue. After 24 h, the eggs were counted (fertility) and transferred to a standard diet followed by incubation for 8 days. Hatched larvae were counted to determine fecundity (Bingsohn et al., 2016).

### Statistics

Statistical analysis was carried out using R (v3.3.2, R Development Core Team, 2008). The survival data were evaluated using Kaplan Meir statistics in the ‘survival’ package (v 2.40.1 Therneau and Grambsch, 2001) and multiple pairwise comparisons among groups were carried out using log-rank tests. *p*-values were adjusted using the ‘holm’ correction method. The fertility and fecundity data were analyzed by one-way analysis of variance (ANOVA) and the ‘holm’ correction method.

## Results

### Identification of Isolates

**FIGURE 1:**
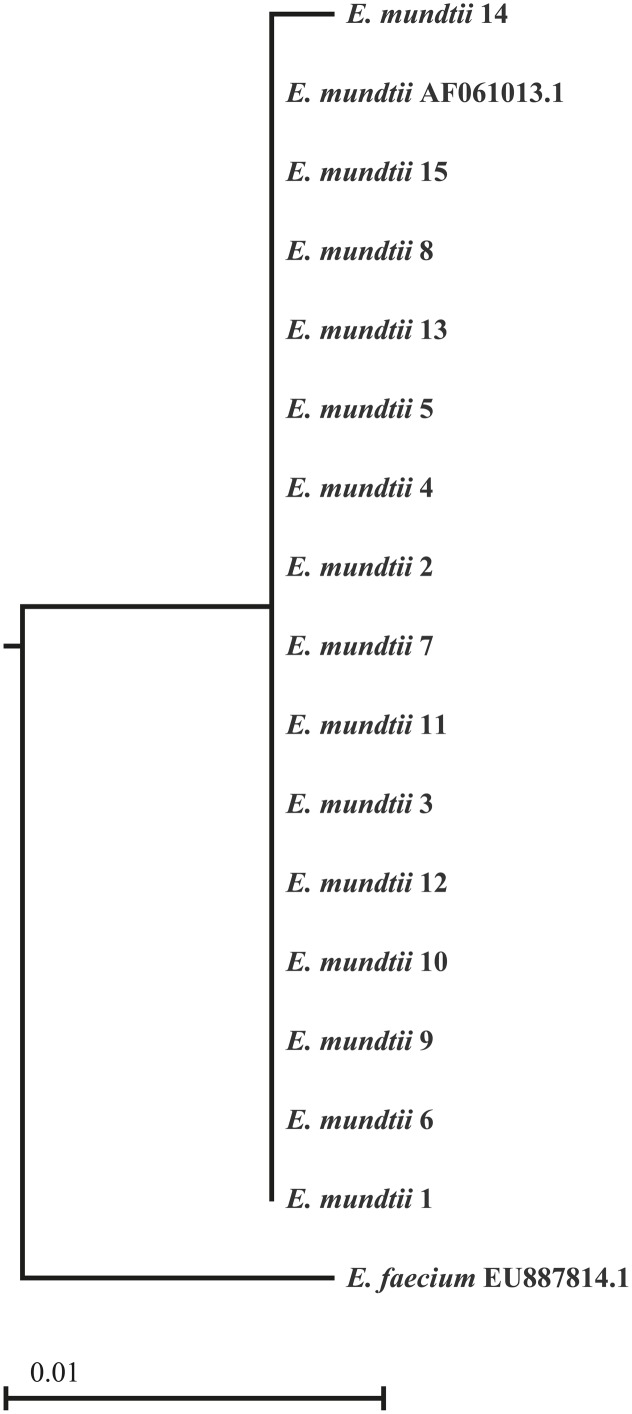
Phylogenetic tree of the 16S rDNA sequences derived from 15 *Enterococcus mundtii* isolates. The sequences from the isolated strains were aligned with two strains identified by their GenBank accession numbers. The scale is based on the bootstrap values from the neighbor-joining method.

BLAST analysis of partial 16S rDNA sequences from 15 isolates showed 100% identity to *E. mundtii*. The phylogenetic relationships shown in **Figure 1** confirm that the isolates are strains of *E. mundtii*.

### Antimicrobial Characterization

An initial screen using the agar spot on lawn method revealed that all 15 isolates showed antimicrobial activity against a broad spectrum of indicator bacteria (**Table 1**). Some isolates also showed activity against entomopathogenic *B. thuringiensis* and *P. entomophila*, as well as human pathogenic bacteria such as *P. aeruginosa* and *S. aureus*. For the further characterization of the antimicrobial compounds, we focused on the CFS of *E. mundtii* isolate 1 using the AWDA (**Table 2**) because this isolate showed the best antimicrobial profile in the agar spot on lawn assay. The crude CFS did not show antimicrobial activity against the indicator bacteria, so we decided to concentrate the CFS by 100-fold. The concentrated CFS was able to inhibit the indicator bacteria to different degrees (**Table 2**). Treatment of the CFS with proteinase K or catalase reduced its antimicrobial activity against some of the indicator bacteria (**Table 2**).

**Table 2:** Agar well-diffusion assay with CFS from *E. mundtii* isolate 1 with different treatments against indicator bacteria.

Indicator strains	Crude	100 × fold	Proteinase K	Catalase	Heated
*Bacillus thuringiensis* (DSM 2046)	0	4.75 ± 0.32	4.23 ± 0.09	4.29 ± 0.12	4.58 ± 0.58
*Escherichia coli* (strain D 31)	0	4.75 ± 0.43	4.81 ± 0.69	4.33 ± 0.27	4.60 ± 0.57
*Klebsiella terrigena* (DSM 2687)	0	6.3 ± 0.17	6.12 ± 0.08	4.73 ± 1.00	6.14 ± 0.26
*Listeria grayi* (DSM 20601)	0	2.25 ± 0.53	1.02 ± 0.15	2.43 ± 0.29	2.55 ± 0.06
*Listeria innocua* (DSM 20649)	0	3.27 ± 0.37	0.56 ± 0.96	2.82 ± 0.13	2.97 ± 0.34
*Listeria seeligeri* (DSM 20751)	0	2.03 ± 1.19	0.97 ± 0.86	2.71 ± 0.37	2.08 ± 1.05
*Micrococcus luteus* (DSM 20030)	0	3.92 ± 0.41	3.68 ± 0.69	3.83 ± 0.46	3.69 ± 0.26
*Pseudomonas aeruginosa* (DSM 50071)	0	4.9 ± 0.22	4.49 ± 0.57	4.07 ± 0.62	4.88 ± 0.39
*Pseudomonas entomophila* (DSM 28517)	0	3.83 ± 0.42	3.68 ± 0.36	3.62 ± 0.31	3.52 ± 0.38
*Salmonella subterranean* (DSM 1620)	0	6.72 ± 0.42	6.39 ± 0.31	6.33 ± 0.49	6.48 ± 0.08
*Staphylococcus aureus* (DSM 2569)	0	0	0	0	0


*The radius of the zone of inhibition is measured in mm. Results are mean values of three replicates with standard deviations.*

### Probiotic Characterization

The cell surface properties of *E. mundtii* 1 isolate are shown in **Table 3**. The isolate showed a high rate of auto-aggregation after 24 h, indicating a significant level of bacterial adhesion to host cells. However, the low level of adhesion to solvents indicated that the surface was hydrophilic. The viability of *E. mundtii* 1 was reduced at pH 2 and slightly reduced at pH 3 (**Table 4**).

**Table 3:** Auto-aggregation rate and hydrophobicity of *E. mundtii* 1.

	*E. mundtii* 1
Auto-aggregation (%) 5 h	12.69 ± 1.28
Auto-aggregation (%) 24 h	89.66 ± 2.83
Hydrophobicity (%)	8.30 ± 9.4


**Table 4:** Effect of low pH on *E. mundtii* 1.

	*E. mundtii* 1
CFU start	8.50 ± 0.07
CFU pH 2 after 3 h	4.11 ± 0.04
CFU pH 3 after 3 h	6.49 ± 0.05
CFU pH 4 after 3 h	8.31 ± 0.037


*Results are in log CFU ml^*-1*^ with SD.*

### Safety Characterization

The microdilution broth assay was used to determine MIC values. *E. mundtii* 1 was susceptible to all the antibiotics listed in **Table 5**. There was no evidence of hemolytic activity on sheep blood agar plated with any of the 15 *E. mundtii* isolates.

**Table 5:** Antimicrobial susceptibility of *E. mundtii* 1.

Antimicrobial agent	MIC (μg ml^-1^)
Ampicillin	<0.25
Ciprofloxacin	<0.25
Erythromycin	<0.25
Fosfomycin	4
Kanamycin	32
Rifampicin	<0.25
Streptomycin	64
Tetracycline	<0.25
Vancomycin	<0.25


### *In Vivo* Characterization

**FIGURE 2:**
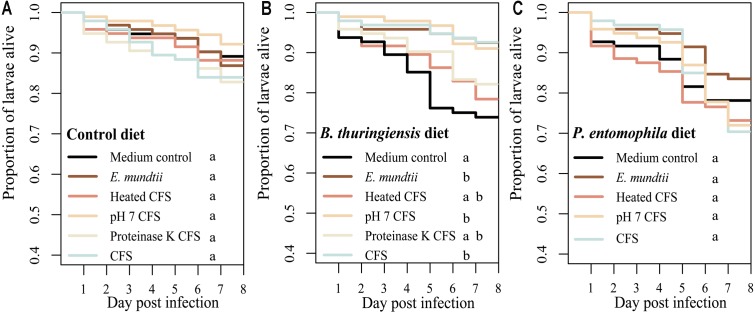
The effect of probiotic diets on *Tribolium castaneum* survival. Survival rates of larvae raised on different probiotic diets based on flour mixed with (i) MRS (control diet), (ii) *E. mundtii*, (iii) CFS pH 7, (iv) CFS treated with proteinase K, (v) CFS heated to 98°C, or (vi) crude CFS. Eight days after exposure to the probiotic diets, larvae (*n* = 96 per treatment) were challenged with **(A)** the control diet, **(B)** a diet spiked with *Bacillus thuringiensis* diet, or **(C)** a diet spiked with *Pseudomonas entomophila*. Statistically significant differences in the treatments are indicated by differing lowercase letters (*p* < 0.05). Precise *p*-values for the multiple comparisons are presented in Supplementary Tables S1–S3.

The potential probiotic effect of the *E. mundtii* 1 isolate (and the corresponding supernatant) was tested by measuring the survival of *T. castaneum* when challenged with a pathogen in the presence or absence of the isolate. There were no significant differences in survival when we compared larvae fed on the control diet with or without an earlier exposure to the probiotic diet (χ^2^ = 5.1, df = 5, *p* = 0.399) (**Figure 2A**) indicating that isolate had no adverse effects on the larvae. In contrast, there was a significant increase in survival rates when we compared larvae fed on the diet containing *B. thuringiensis* with or without an earlier exposure to the probiotic diets (χ^2^ = 24.8, df = 5, *p* < 0.0001) (**Figure 2B**). The above probiotic effect of the CFS was reduced by treatment with proteinase K or by heating to 98°C. In contrast to the diets spiked with *B. thuringiensis*, there was no significant difference in survival rates when we compared larvae fed on the diet containing *P. entomophila* with or without an earlier exposure to the probiotic diet (χ^2^ = 4, df = 4, *p* = 0.403) (**Figure 2C**). The precise *p*-values from multiple pairwise comparisons of the Kaplan Meir curves with ‘holm’ correction are presented in Supplementary Tables S2–S4.

**FIGURE 3:**
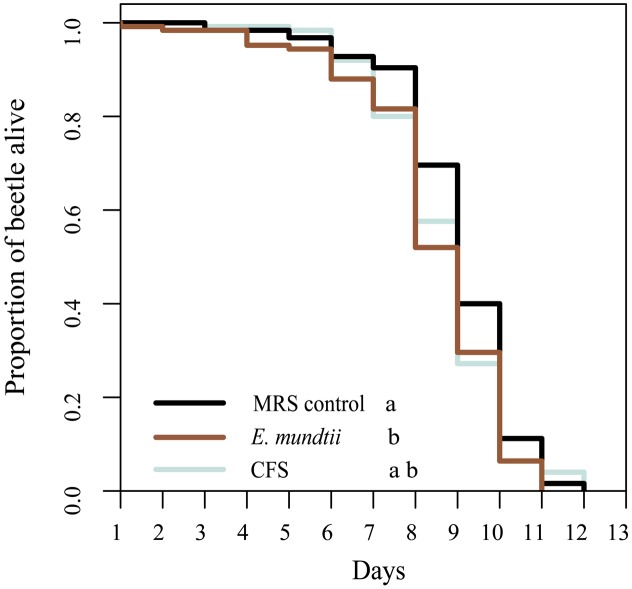
Longevity of *T. castaneum*. Larvae were raised on different probiotic diets, and age controlled adult beetles were maintained on the same diets. Survival was determined by performing a thermotolerance assay (five replicates with 25 beetles of mixed sex). Statistically significant differences between treatments are indicated by differing lowercase letters (*p* < 0.05). Precise *p*-values for the multiple comparisons are presented in Supplementary Table S4.

The potential impact of the *E. mundtii* isolate on the longevity of *T. castaneum* was determined using a thermotolerance assay. We observed a significantly shorter lifespan when beetles were reared on diets containing *E. mundtii* (χ^2^ = 6.4, df = 2, *p* = 0.0398) (**Figure 3**). However, there were no significant differences in longevity among beetles fed on the CFS diet, the MRS control diet or the *E. mundtii* diet (Supplementary Table S5). We also investigated the influence of the probiotic diet on the fitness (fertility and fecundity) of *T. castaneum* and found that the number of eggs differed significantly in a diet-dependent manner (ANOVA df = 2; *F* = 17,163; *p* < 0.001) (Supplementary Figure S1). Multiple pairwise comparisons among the diet groups showed that beetles maintained on the probiotic *E. mundtii* diet laid significantly fewer eggs than beetles on the CFS and control diets (*p* < 0.001). However, there was no significant difference in fecundity among the diet groups (ANOVA df = 2; *F* = 0.0327; *p* = 0.968).

## Discussion

We have shown that probiotic bacteria can be isolated from the feces of storage food insects. Based on the analysis of 16S rDNA sequences we isolated several new strains of *E. mundtii*, and characterized their probiotic profiles as well as their broad antimicrobial activity against Gram-positive and Gram-negative bacteria. We also show the potential for *T. castaneum* as a model system for screening of such probiotic bacteria *in vivo*.

The screen for antimicrobial activity revealed a broad spectrum of activity in all 15 isolates we tested. However, we were unable to detect antimicrobial activity in the crude CFS, in contrast to previous studies of *E. mundtii*, which reported a broad spectrum of antimicrobial activity also in the crude CFS (Zendo et al., 2005; Schelegueda et al., 2015). We were able to detect a similar antimicrobial profile in the agar spot on lawn assay and CFS only when the latter was concentrated by 100-fold. *E. mundtii* belongs to the order lactobacillales, whose members are known to produce a variety of heat-stable bacterocins that are sensitive to proteinase K (Franz et al., 2007). However, proteinase K and catalase treatments reduced the antimicrobial activity of only a few of our isolates, and heat treatment had no impact *in vitro*. By adjusting the pH of the extract, we ruled out the possibility that antimicrobial activity was based on organic acids. The major antimicrobial compounds in the isolates are therefore heat stable and resistant to proteinase K, as previously reported (Silva et al., 1987; Ouzari et al., 2008). Further testing is required to identify and characterize the antimicrobial compounds in detail. In many screens for probiotic bacteria, only the crude CFS is tested for antimicrobial activity, which may lead to false negative results given that many genes remain silent under standard laboratory cultivation conditions and the corresponding compounds may not be synthesized (Nett et al., 2009; Seyedsayamdost, 2014; Rutledge and Challis, 2015).

To further characterize the probiotic profile of one of our isolates we investigated the surface properties of *E. mundtii* isolate 1. This isolate showed a high level of auto-aggregation after 24 h and a low level of solvent adhesion. Auto-aggregation is related to the ability of bacterial cells to adhere to epithelial cells and form colonies (Kos et al., 2003), which is a prerequisite for probiotic bacteria because this is how they colonize the gut. The low solvent adhesion indicates the presence of a hydrophilic cell surface based on polysaccharides (Collado et al., 2007, 2008). *E. mundtii* 1 isolate was able to survive in a low-pH environment, making the isolate resistant to the harsh conditions in the digestive system (Conway et al., 1987; Jena et al., 2013). It is also important to evaluate the safety of probiotic bacteria, especially in the genus *Enterococcus*, because the closely related species *E. faecalis* possesses hemolytic activity (De Vuyst et al., 2003). We could not detect hemolytic activity in any of our isolates. There are also concerns that Enterococci may be able to transfer antibiotic resistance genes (Palmer et al., 2010), but *E. mundtii* isolate 1 was susceptible to all the antibiotics we tested.

*In vitro* assays allow the initial preselection of potentially probiotic strains, but *in vivo* testing is also necessary to check for systemic interactions (Papadimitriou et al., 2015). We introduced the model organism *T. castaneum* as a novel *in vivo* probiotic screening platform. *T. castaneum* is an established model organism that can be used for infection assays and has the capacity for immune priming (Roth et al., 2010; Knorr et al., 2015; Futo et al., 2016). The feeding of *T. castaneum* larvae with either *E. mundtii* 1 or the corresponding CFS protected them against the entomopathogen *B. thuringiensis* but not against *P. entomophila*. A similar result was reported for *C. elegans*, i.e., feeding with Gram-positive probiotics resulted in protection only against Gram-positive pathogens and not against Gram-negative pathogens (Kim and Mylonakis, 2012). The crude CFS showed no *in vitro* antimicrobial activity but nevertheless resulted in a protective function *in vivo*, indicating that the probiotic properties are not based on antimicrobial activity. Both *E. mundtii* and *B. thuringiensis* are Gram-positive bacteria, which should activate the same host immune responses upon contact, in contrast to Gram-negative bacteria as *P. entomophila* (Lemaitre and Hoffmann, 2007; Yokoi et al., 2012). Although the *in vivo* protective effect of the CFS was reduced by heating to 98°C or treatment with proteinase K, the loss of activity was not significant. This may indicate that the protective function of the CFS is based on bacterocins, which are thought to act as signaling peptides for communication with other bacteria or the host immune system (Cotter et al., 2013). The diverse class of bacterocins includes proteinase K-sensitive as well as heat-labile compounds (Yang et al., 2014). Alternatively, Gram-positive cell wall compounds such as peptidoglycans and lipoteichoic acid are thought to activate the immune system (Leulier et al., 2003; Rao and Yu, 2010). Probiotic bacteria are widely used in aquaculture as food supplements and for the white leg shrimp *Litopenaeus vannamei*, where the supernatant of a probiotic bacterial culture also protects the hosts from pathogens and induces specific immune system genes (Sha et al., 2016). Further research is required to investigate the protective function of the supernatant in more detail, and to determine whether the protective function involves the modulation of the immune system or alters the dynamics of the larval gut microbiota.

Although, our isolate increased host resistance toward an entomopathogen there was a trade-off in terms of beetle fitness. Certain probiotic bacteria have been shown to increase the lifespan of *C. elegans* (Grompone et al., 2012; Park et al., 2015) but our isolate had the opposite effect on *T. castaneum*. Furthermore the fertility of the beetle was also reduced following treatment with the *E. mundtii* isolate. One potential explanation for this phenomenon is the recently observed translocation of bacteria from the *T. castaneum* gut to the eggs, where they elicit an innate immune response that triggers a fitness penalty (Knorr et al., 2015). The potential for such adverse effects highlights the importance of *in vivo* assays for the characterization of probiotic bacteria.

Our results confirm that *T. castaneum* is suitable as an alternative model invertebrate for pre-screening in probiotic research, for human application pending on further confirmation in the mouse model. The short generation time and longevity of *T. castaneum* makes it particularly suitable for long-term studies of probiotics, including potential transgenerational effects and the influence of probiotics on fitness parameters. Here, *T. castaneum* also becomes of interest as test organism with respect to edible insects being closely related to *Tenebrio molitor*, the most promising candidate in mass rearing of insects for feed and food currently (Grau et al., 2017). Our *E. mundtii* isolate showed beneficial probiotic properties *in vitro* and partly also *in vivo*, suggesting that the feces of insect food pests could be a good source for probiotic bacteria in the future.

## Author Contributions

Planned and conceived the experiments: TG and GJ. Performed the experiments: TG. Analyzed the experiments: TG and GJ. Drafted the manuscript and contributed to the data interpretation: TG, AV, and GJ. All authors read, critically revised and approved the final manuscript.

## Conflict of Interest Statement

The authors declare that the research was conducted in the absence of any commercial or financial relationships that could be construed as a potential conflict of interest.
