# Supplementary material for: Probiotic Enterococcus mundtii Isolate Protects the Model Insect Tribolium castaneum against Bacillus thuringiensis
Source: Front Microbiol. 2017 Jul 7;8:1261. doi: 10.3389/fmicb.2017.01261 (PMC5500611; doi:10.3389/fmicb.2017.01261)
Supplement: Supplementary file 1 [file Data_Sheet_1.docx]

Supplement to

Probiotic *Enterococcus mundtii* isolate protects the model insect *Tribolium castaneum* against *Bacillus thuringiensis*

Thorben Grau^1^, Andreas Vilcinskas^1,2^, Gerrit Joop^1^

^1^Institute for Insect Biotechnology, Justus-Liebig-University Giessen, Giessen, Germany

^2^Department of Bioresources, Fraunhofer Institute for Molecular Biology and Applied Ecology, Giessen, Germany

***Correspondence:** Gerrit Joop, Institute of Insect Biotechnology, Justus-Liebig University Giessen, 35392 Giessen, Heinrich Buff Ring 29-32, Germany

[Gerrit.Joop@agrar.uni-giessen.de](mailto:Gerrit.Joop@agrar.uni-giessen.de)

Table S1 GenBank accession numbers

| Isolate | GeneBank ID |
| --- | --- |
| *E. mundtii* 1 | MF136790 |
| *E. mundtii* 2 | MF136791 |
| *E. mundtii* 3 | MF136792 |
| *E. mundtii* 4 | MF136793 |
| *E. mundtii* 5 | MF136794 |
| *E. mundtii* 6 | MF136795 |
| *E. mundtii* 7 | MF136796 |
| *E. mundtii* 8 | MF136797 |
| *E. mundtii* 9 | MF136798 |
| *E. mundtii* 10 | MF136799 |
| *E. mundtii* 11 | MF136800 |
| *E. mundtii* 12 | MF136801 |
| *E. mundtii* 13 | MF136802 |
| *E. mundtii* 14 | MF136803 |
| *E. mundtii* 15 | MF136804 |

Table S2 Multiple pairwise comparison of control challenge diet. P- values of the multiple pairwise comparison with ‘holm’ correction of the Kaplan-Meier survival curves presented in Figure 2A

| Control challenged | MRS | *E. mundtii* | Heated | pH 7 | Proteinase K |
| --- | --- | --- | --- | --- | --- |
| *E. mundtii* | 1 |  |  |  |  |
| Heated | 1 | 1 |  |  |  |
| pH 7 | 1 | 1 | 1 |  |  |
| Proteinase K | 1 | 1 | 1 | 0.733 |  |
| CFS | 1 | 1 | 1 | 1 | 1 |

Table S3 Multiple pairwise comparison of *B. thuringiensis* challenge diet. P- values of the multiple pairwise comparison with ‘holm’ correction of the Kaplan-Meier survival curves presented in Figure 2B

| *B. thuringensis* challenged | MRS | *E. mundtii* | Heated | pH 7 | Proteinase K |
| --- | --- | --- | --- | --- | --- |
| *E. mundtii* | 0.011 |  |  |  |  |
| Heated | 1 | 0.079 |  |  |  |
| pH 7 | 0.019 | 1 | 0.147 |  |  |
| Proteinase K | 0.938 | 0.314 | 1 | 0.475 |  |
| CFS | 0.009 | 1 | 0.076 | 1 | 0.314 |

Table S4 Multiple pairwise comparison of *P. entomophila* challenge diet. P-values of the multiple pairwise comparison with ‘holm’ correction of the Kaplan-Meier survival curves presented in Figure 2C

| *P. entomophila* challenged | MRS | *E. mundtii* | Heated | pH 7 | Proteinase K |
| --- | --- | --- | --- | --- | --- |
| *E. mundtii* | 1 |  |  |  |  |
| Heated | 1 | 0.851 |  |  |  |
| pH 7 | 1 | 0.807 | 1 |  |  |
| CFS | 1 | 0.859 | 1 | 1 | 1 |

Table S5 Multiple pairwise comparison of longevity of *T. castaneum*. P-values of the multiple pairwise comparison with ‘holm’ correction of the Kaplan-Meier survival curves presented in Figure 3

|  | MRS | *E. mundtii* |
| --- | --- | --- |
| *E. mundtii* | 0.026 |  |
| CFS | 0.252 | 0.348 |

*
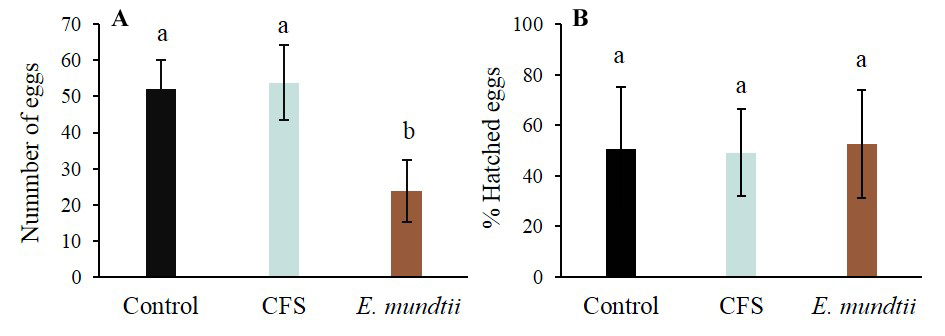
*

Figure S1 Fitness parameters of *T. castaneum*. Larvae were raised on different probiotic diets and age controlled virgin adult beetles (5 each sex) were set for 24 h mating. (A) Eggs (fertility) were counted after 24 hours. (B) Hatching rate (fecundity) was counted after 8 days. Five replicates each treatment. Statistical analysis was done by ANOVA with holm correction. Statistical significant differences in the treatments are indicated by differing lowercase letters (p < 0.05).
